# Synthesis of Phosphonated Carbon Nanotubes: New Insight into Carbon Nanotubes Functionalization

**DOI:** 10.3390/ma14112726

**Published:** 2021-05-21

**Authors:** Małgorzata Nadolska, Marta Prześniak-Welenc, Marcin Łapiński, Kamila Sadowska

**Affiliations:** 1 Institute of Nanotechnology and Materials Engineering, Gdansk University of Technology, 80-233 Gdansk, Poland; malgorzata.nadolska@pg.edu.pl (M.N.); marwelen@pg.edu.pl (M.P.-W.); marcin.lapinski@pg.edu.pl (M.Ł.); 2 Institute of Biocybernetics, Biomedical Engineering of the Polish Academy of Sciences, 02-109 Warsaw, Poland

**Keywords:** carbon nanotubes, chemical functionalization, phosphorus containing groups, phosphonates, free-radical reactions

## Abstract

Carbon nanotubes were successfully functionalized for the first time in a free radical phosphonylation reaction. Three synthetic protocols were proposed. Carbon nanotubes and diethylphosphite reacted in the presence of known radical initiator, such as azobisisobutyronitrile, single electron oxidant—Mn(OAc)_3_, or under UV radiation. The functionalized material was fully characterized by means of spectroscopic methods, together with microscopic, surface area and thermogravimetric analyses. UV-illumination was found to be the most effective approach for introducing phosphonates onto carbon nanotubes. X-ray photoelectron spectroscopy analysis showed 6% phosphorus in this sample. Moreover, the method was performed at room temperature for only one hour, using diethylphosphite as a reactant and as a solvent. The functionalized carbon nanotubes showed an improved thermal stability, with a decomposition onset temperature increase of more than 130 °C. This makes it very promising material for flame retarding applications.

## 1. Introduction

Carbon nanotubes (CNTs) receive a great deal of attention due to their impressive properties and variety of applications. Owing to their large specific surface area and high electrical conductivity, CNTs are promising candidates for sensors [1,2], energy storage materials [3,4], and catalyst supports [5,6]. A well-developed surface combined with good chemical stability also makes them effective pollutant adsorbents [7,8] and excellent drug and gene carriers [9,10]. However, in most cases, CNTs cannot be used in their pristine form and their surface properties usually need to be tailored to specific application.

Surface functionalization is a powerful tool that allows to adjust the physical and chemical properties of CNTs, and, as a consequence, increase their processability and applicability. There are many approaches that have been applied for the functionalization of CNTs, including covalent and noncovalent modifications using chemical and physical methods (plasma treating, thermal annealing) [11,12,13,14]. One key area of research deals with the introduction of different functional groups onto the surface of CNTs. The most common are definitely oxygen functional groups (e.g., hydroxyl, carboxyl, carbonyl); however, many examples of CNTs with nitrogen (e.g., amine, amide) and sulfur-containing (e.g., thiol, sulfonic) functional groups can also be found in the literature [15,16,17,18]. Contrary to the examples mentioned above, phosphorus-containing groups, especially those with a phosphorus atom attached directly to the carbon skeleton, are rarely reported. At the same time, organic compounds containing C-P bonds find a number of applications, such as corrosion inhibitors [19,20], detergents [21], flame retardants [22,23], and others. It is also well known that such compounds possess a high affinity towards various metal ions and can be used in waste water treatment [24,25]. C–P bonds are also widely found in many biologically active compounds, with applications ranging from enzyme inhibitors to bone-seeking pharmaceuticals [26,27]. The vast majority of literature reports include noncovalent approaches, e.g., π–π stacking interactions between naphthalen-1-ylmethylphosphonic acid and CNTs [28,29] or the attachment of phosphonates via linkage (e.g., amide or carbonyl bonds [30,31,32,33,34]). Such connections are prone to hydrolysis and can suffer from insufficient thermal stability. In the literature, there are only a few examples described in which phosphorus-containing groups are directly connected to the carbon of nanotubes. Dehghani and co-workers used chemical vapor deposition method to obtain multi-walled carbon nanotubes functionalized with phosphonic acid [35,36]. The functionalization degree of such a product was high (11.65%), but the synthesis protocol required a high temperature (1300 °C) and a special system to guaranteed a controlled atmosphere and proper gas flow. CNTs functionalized with triphenylphosphine have also been reported [37,38,39]. The conversion of the carboxyl groups present in CNTs into phosphonic ones was proposed by two groups to produce bis-phosphonic derivatives [40,41].

In the present work, we grafted phosphorus-containing groups directly to the carbon skeleton in a free-radical phosphonylation reaction. It should be noted that the reaction of phosphorus radicals with sp^2^ carbon to form C–P bond compounds is known from classical organic chemistry [42]. However, studies on the reaction of phosphorus radicals with carbonaceous nanostructures are rare. To the best of our knowledge, reported works include only fullerenes [43,44]. Herein, we adopt this method for multi-walled carbon nanotubes (MWCNTs). Moreover, we proposed two new protocols, in which the formation of phosphorus-centred free radicals was thermally generated in the presence of an initiator (azobisisobutyronitrile (AIBN)) or by means of UV-irradiation, respectively. The functionalized MWCNTs were thoroughly characterized using spectroscopic (Fourier-Transform Infrared Spectroscopy (FTIR), X-ray Photoelectron Spectroscopy (XPS) andEnergy Dispersive X-ray spectroscopy (EDX) and microscopic methods (Scanning Electron Microscopy (SEM)). Furthermore, thermal stability and surface area (adsorption–desorption nitrogen isotherms) analyses have been carried out.

## 2. Materials and Methods

Multi-walled carbon nanotubes (MWCNTs, average outer diameter: 8–15 nm, purity >95 wt%) were ordered from Cheaptubes Inc., Grafton, VT, USA. Diethyl phosphite ((C_2_H_5_O)_2_P(O)H), manganese (III) acetate dihydrate (Mn(OAc)_3_·2H_2_O), azobisisobutyronitrile (AIBN) and 1,2-dichlorobenzene, tetrahydrofuran (THF) and N,N-dimethylformamide (DMF) were purchased from Acros Organics, Antwerp, Belgium. The chemicals were of analytical grade and were used without further purification.

Multi-walled carbon nanotubes were functionalized through free-radical reactions. In all syntheses, the same amount of carbon material (100 mg) and diethyl phosphite (3 mL), which acted as a phosphate group source, were used. Reactions were carried out in the presence of known radical initiators, such as Mn(OAc)_3_ and AIBN, or under UV radiation. The UV-promoted reaction was carried out in a photoreactor prototype designed by Dariusz Wysiecki MSc., Eng. and constructed in cooperation with the Enviklim Company (Gdańsk, Poland). The reactor was equipped with 3 UVA diode arrays: 2xUV-D6565-4LED, 40 W and 1xUV-D6565-15LED, 150 W (Enviklim Company, Gdańsk, Poland) with a 365–370 nm wavelength [45,46]. Detailed conditions are summarized in Table 1. Afterwards, products were washed with DMF and THF, and dried under reduced pressure. The obtained functionalized MWCNTs were labelled as MWCNT–Phos–Mn and MWCNT–Phos–AIBN, in the case of using Mn(OAc)_3_ and AIBN, respectively, and MWCNT–Phos–UV when UV-irradiation was used.

### Characterization

The morphologies of the samples were studied with a scanning electron microscope (ESEM Quanta Feg 250, FEI, Waltham, MA, USA). Fourier transform infrared (FTIR) spectra were collected on a Perkin Elmer Frontier spectrophotometer (Waltham, MA, USA) in the range of 500–4000 cm^−1^. Measurements were made in transmittance mode and the potassium bromide pellet method was used. X-ray diffraction patterns were collected on a Bruker D2 Phaser 2nd generation diffractometer with CuK_α_ radiation (λ = 1.5404 Å) with2θ ranging from 5 to 70°. The Raman spectra were obtained with an integrated confocal micro-Raman system with a LabRamAramis (Horiba Jobin Yvon, Tokyo, Japan) 460-mm spectrometer equipped with a confocal microscope. The excitation source was a diode pumped solid state (DPSS) laser emitting light at 632.8 nm. The surface chemical composition was studied using an Omicron NanoTechnology spectrometer (ScientaOmicron, Uppsala, Sweden) with Mg Kα as an excitation source. The binding energies were corrected using the background C1s (285.0 eV) line as a reference. XPS spectra were analyzed with Casa-XPS software (Casa Software Ltd., ver. 2.3.23., Devon, UK) using Shirley background subtraction and Gaussian–Lorentzian curve as a fitting algorithm. Thermogravimetric analysis (TGA) was performed with a Netzsch STA 449 F1 (Netzsch, Selb, Germany). Experiments were carried out with a heating rate 10 °C/min from 40 °C to 900 °C under an argon atmosphere and under synthetic air. The N_2_ adsorption–desorption isotherms were measured on a NOVAtouch™ 2 surface area analyser (Quantachrome Instruments, Boynton Beach, FL, USA) at 77 K. Prior to the measurements, samples were degassed under vacuum at 40 °C for 12 h. Specific surface area was calculated from the Brunauer–Emmett–Teller (BET) linear equation in the range of 0.1–0.3 relative pressure. The correlation coefficient of the linear regression was not less than 0.999. 

## 3. Results and Discussion

In the present work, we present three ways of bonding of phosphorus-containing groups directly to the carbon skeleton of nanotubes by using phosphorus-centred radicals. It should be noticed, that the reaction of phosphorus-centred radicals with sp^2^ carbon to form C–P bonds compounds is known from classical organic chemistry [42]. Homolytic cleavage of P–H bonds serves as a major approach for the generation of phosphorus-centred radicals. Phosphonyl radicals are the most popular for use in the generation of C–P bonds with alkenes, alkynes, arenes, and heteroarenes [47,48], and they can be generated from corresponding phosphonates under appropriate radical initiation conditions [49,50,51]. Generally, phosphorus-centred radicals can be generated via thermolysis or photolysis, and/or in the presence of well-known initiators, such as peroxides, azo compounds, or photo radical initiators. The reactions with single electron oxidants, such as cerium ammonium nitrate, Mn(OAc)_2_/O_2_ or Mn(OAc)_3_, and AgOAc/K_2_S_2_O_6_ have also been reported.

**Figure 1:**
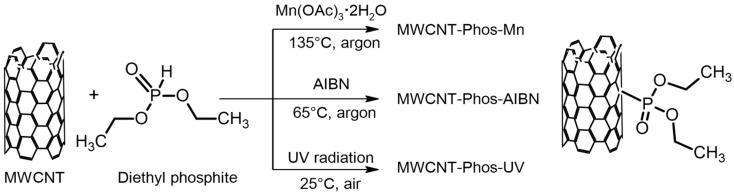
Three pathways leading to phosphonated MWCNTs.

Interestingly, since the pioneering work of Jeffrey L. Bahr and James M. Tour [52], carbon-centred radicals have been frequently used for carbon nanostructures functionalization, while there is only one example in the literature describing the reaction of phosphorus-centred radicals with carbon nanomaterials. Wang and co-workers reported the Mn(OAc)_3_-promoted reaction of C60 with phosphonate esters or phosphine oxide. Depending on the reaction conditions (i.e., molar ratio of the substrates) they obtained three different types of phosphorylated fullerenes, that is, singly bonded fullerene dimers, hydrofullerenes, and acetoxylated fullerenes [43,44]. Based on knowledge from organic chemistry and our experience in the free-radical functionalization of carbon nanotubes using diazonium salts [39,53,54,55,56,57], we proposed three synthesis pathways for covalent functionalization of MWCNTs using diethyl phosphite, which are presented in Figure 1.

**Figure 2:**
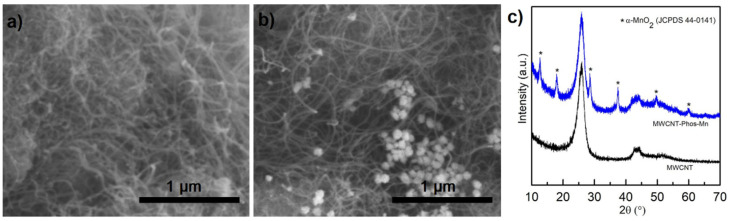
SEM images of MWCNT (**a**) and MWCNT–Phos–Mn (**b**) and their XRD patterns (**c**).

The first synthesis was following the work of Wang; however, using carbon nanotubes in the place of fullerenes. The reaction was performed under argon, in 1,2-dichlorobenzene with Mn(OAc)_3_ as a single electron oxidant, promoting (EtO)_2_P(O)· formation from (EtO)_2_P(O)H at an elevated temperature (135 °C). The functionalization was successful; however, a detailed analysis of the obtained material revealed the presence of a by-product, which was impossible to remove. Figure 2a,b shows SEM images of MWCNTs before and after functionalization. It can be clearly seen that the carbon material is decorated with particles, which were not present in the pristine nanotubes. XRD study proved our assumption, that it was MnO_2_ formed as a by-product of Mn(OAc)_3_ decomposition. A comparison of diffractograms recorded for pristine MWCNTs and the MWCNT–Phos–Mn sample is presented in Figure 2c. Typical (002) and (100) reflexes of MWCNT are present at 26° and 43° in both diffractograms; however, additional reflexes are visible only for the functionalized sample. The reflexes marked with asterisks refer to an α-MnO_2_ crystal structure (Joint Committee on Powder Diffraction Standards (JCPDS)44-0141). Spectroscopic and thermal analyses revealed other differences and they will be discussed later in comparison to the material obtained using the two other proposed synthesis pathways.

As the obtained material, although interesting, was not what we expected, another synthetic protocol was proposed. In a second approach, thermal decomposition of azobisisobutyronitrile (AIBN) served as a free radical reaction initiator to further produce phosphonyl radicals from diethyl phosphite. The sample was heated for 5 h under argon at 65 °C in diethylphosphite, which served as a reagent and a solvent. The obtained sample was denoted as MWCNT–Phos–AIBN. The final method was based on photo-induced generation of phosphonyl radicals from diethyl phosphite in a solvent-less reaction carried out for 1 h at RT under UV light illumination (P = 230 W; λ = 365–370 nm). The sample was labelled as MWCNT–Phos–UV.

**Figure 3:**
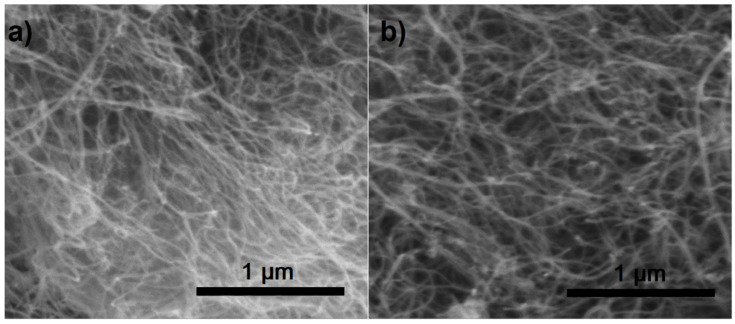
SEM images of MWCNT–Phos–AIBN (**a**) and MWCNT–Phos–UV (**b**).

Similar to the MWCNT–Phos–Mn sample, the other two were also observed under SEM. The obtained images can be seen in Figure 3. No significant structural changes were visible for the studied samples in reference to the pristine nanotubes. However, large area imaging presented a higher purity of functionalized samples in terms of a lower presence of amorphous carbon. It is frequently reported, that nanotubes are purified during a functionalization reaction and washing after synthesis [58,59].

**Figure 4:**
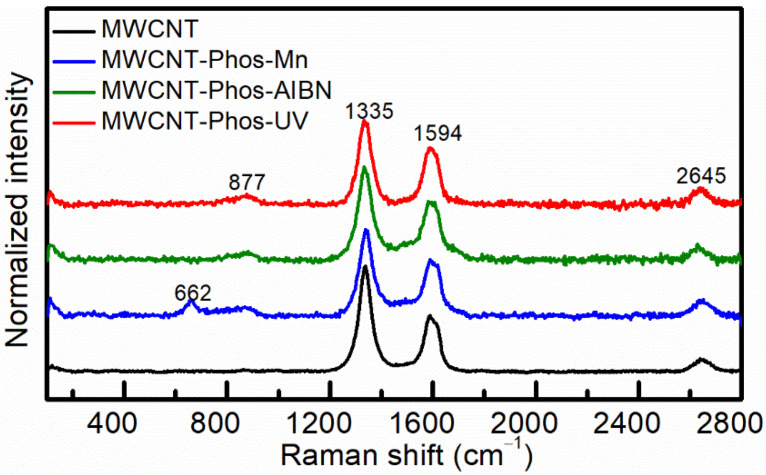
Raman spectra of pristine and functionalized MWCNTs.

Pristine and functionalized MWCNTs were characterized using Raman, FTIR, and XPS spectroscopy. Raman spectra of MWCNTs samples recorded at a633 nm excitation wavelength are presented in Figure 4. Raman spectra of the pristine nanotubes are in accordance with reported data for this type of material and excitation energy [60,61,62]. Typical D and G bands were recorded for all samples. A comparison of Raman mode positions, full width at half maximum (FWHM), and I_D_/I_G_ ratio are presented in Table 2. The decrease inthe *I*_D_/*I*_G_ ratio was ascribed to the removal of amorphous carbon and the most defective MWCNTs during washing after functionalization [63,64]. Both the D band and G band of functionalized MWCNTs are widened, indicating the introduction of functional groups into the nanotube structures [65]. Moreover, the G bands of MWCNT–Phos–AIBN and MWCNT–Phos–UV are shifted to a lower wavenumber. Similar results were obtained by Sunet al. [66] and described as evidence of the formation of C-P bonds.

After functionalization, a new band at 877 cm^−1^ appeared in the spectra of functionalized samples, being most evident for MWCNT–Phos–AIBN and MWCNT–Phos–UV. This can be ascribed to a superposition of bands, referring to C–O–P, C–P and C–C–O stretching. The 662 cm^−1^ band, visible only in the MWCNT–Phos–Mn spectrum is additional evidence of the presence of MnO_2_ in this sample. According to the literature, the spectrum of α-MnO_2_ exhibits peaks in the region of 400–800 cm^−1^, which are ascribed to the stretching mode of the MnO_6_ octahedra. The highest band present in standard α-MnO_2_ around 650 cm^−1^ could be attributed to the symmetric stretching vibration (Mn–O) of the MnO_6_ groups. In MWCNT–Phos–Mn the band is observed at 662 cm^−1^ and its shift to higher wavenumbers may be due to the presence of carbon material in the sample. A similar behaviour was observed for MnO_2_/graphene composites by Liu et al. [67].

**Figure 5:**
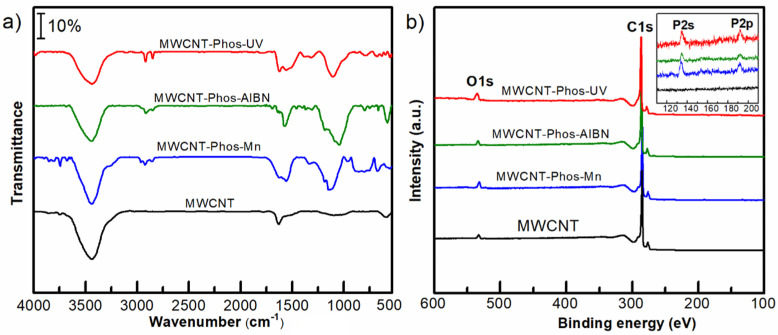
FTIR (**a**) and XPS survey (**b**) spectra of pristine and functionalized MWCNTs.

The presence of functional groups was evidenced by FTIR and XPS spectroscopy. The FTIR spectra of functionalized nanotubes in comparison to pristine MWCNTs are presented in Figure 5a. In the spectrum of pristine MWCNTs, a small band originating from carbon–carbon bond vibrations is visible, next to well-pronounced water infrared bands at 3490 and 1630 cm^−1^ [68]. For all functionalized samples, bands at 2914 and 2854 cm^−1^ referring to C–H stretching in the ethoxy group of phosphonate are clearly visible. In the lower frequency region, C–P, P–O and P=O bands are present at 1450, 1180 and 1040 cm^−1^, respectively [69,70]. In the MWCNT–Phos–Mn sample, multiple bands in the range of 900–600 cm^−1^, resulting from Mn–O–Mn stretching are observed. This is additional evidence of the presence of MnO_2_ by-product in this sample [71].

**Figure 6:**
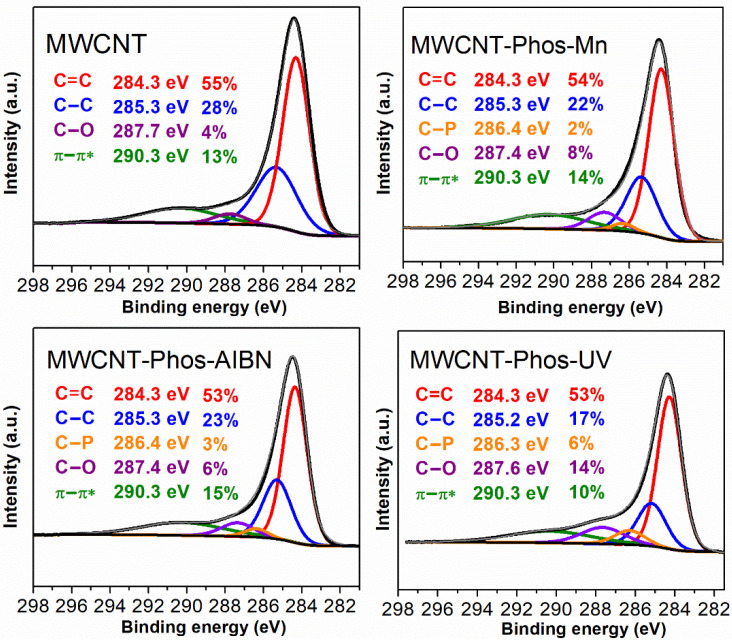
High resolution XPS spectra of C1s with peak positions and calculated atomic percentages.

The functionalization of carbonaceous material with phosphonates was additionally proved using X-ray photoelectron spectroscopy (XPS). The survey spectra of all samples are presented in Figure 5b. It can be seen, that the C to O ratio decreased in the functionalized samples and was lowest in the case of MWCNT–Phos–UV. This indicates, that oxygen content increased due to the introduction of −P(O)(OEt)_2_ groups and the highest functionalization was obtained in the UV-promoted reaction. The inset in Figure 6 shows magnified P 2s and P 2p energy regions. Again, the highest signals were obtained for the MWCNT–Phos–UV sample, while pristine MWCNT did not contain phosphorus. Moreover, grafting of phosphorous-containing groups onto the MWCNT surface was confirmed by the deconvolution of high resolution C 1s spectra and analysis of carbon binding states (Figure 6). Pristine MWCNTs revealed four components C=C (284.3 eV), C–C (285.3 eV), C–O (287.7 eV), and π–π* (290.3 eV), indicating the presence of amorphous carbon and a small amount of oxygen contamination in the sample [72,73]. After functionalization, a new peak appeared at 286.4 eV, which can be assigned to the C–P [32] and is evidence of direct bonding between the carbon skeleton and the functional group. In addition, an increase in C–O peak was observed due to the presence of C–O–P bonds from the introduced group. The highest phosphorus content, ca. 6% at. was recorded for MWCNT–Phos–UV sample, which is in agreement with data obtained in other studies. 

**Figure 7:**
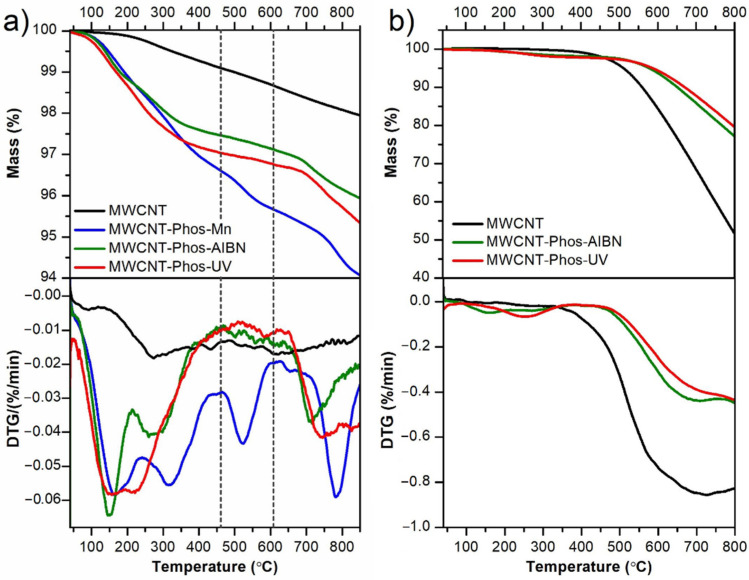
(**a**) TG (upper) and DTG (lower) curves recorded for pristine and functionalized MWCNTs in argon; (**b**) TG curves of MWCNT, MWCNT–Phos–AIBN and MWCNT–Phos–UV registered in synthetic air.

Thermogravimetric analysis conducted in an argon environment revealed differences in the thermal stabilities of the studied samples (Figure 7a). The highest mass loss in the studied temperature range was observed for the MWCNT–Phos–Mn sample. The characteristic steps for the decomposition cascade: MnO_2_ → Mn_2_O_3_ → Mn_3_O_4_ were observed in the thermogravimetric (TG) curve, together with the corresponding minima (520, 788 °C) in the derivative thermogravimetric (DTG) curve [74]. Unfortunately, because of the presence of MnO_2_ in this functionalized MWCNT sample, it was not possible to determine the number of functional groups introduced during synthesis. The TG curves for the two other samples were similar, showing, however, a higher functionalization degree for MWCNT–Phos–UV. The lowest contamination of the MWCNT–Phos–UV sample with amorphous carbon was also proved by this study. The comparison of the observed mass loss for all samples is given in Table 3. The obtained mass loss for the MWCNT–Phos–UV, due to the decomposition of the functional groups, slightly exceeded 3%. This quite-low mass loss can be justified by the flame-retarding properties reported for carbon materials with phosphorus-containing groups [59,75,76]. When phosphonated, carbon nanotubes are heated and a char layer is formed on the surface, which shields the material, preventing the formation of volatile moieties. Therefore, the recorded mass loss during TG analysis cannot be directly connected with the number of phosphonic groups attached to the carbon skeleton. To further prove this assumption, MWCNT–Phos–UV and MWCNT–Phos–AIBN were also studied under oxidative conditions. TG curves recorded in air are presented in Figure 7b. Compared to the pristine MWCNTs, functionalized samples show better thermal stability and higher char yield. It is worth noting, that the experiments were conducted in partial oxygen to be closer to environmental conditions. Therefore, incomplete combustion of CNTs and functionalized CNTs can be read out of the TG curves (Figure 7b). A similar behavior was observed by Mahajan et al. [77]. They observed ca. 50%-mass loss of CNTs heated under partial oxygen with a heating rate of 5 °C/min, which is in line with our observations. Incorporation of phosphorus-containing groups results in an increase in the decomposition onset temperature, from 527 °C for MWCNTs to 639 °C and 652 °C for MWCNT–Phos–AIBN and MWCNT–Phos–UV, respectively. In addition, the first derivative of the TG curves (DTG, Figure 7b) revealed that the decomposition rate is almost two times slower for functionalized MWCNTs. The above results indicate that even a low percentage of functionalization significantly improves the thermal properties of MWCNTs and makes them promising materials as flame retarding additives.

**Figure 8:**
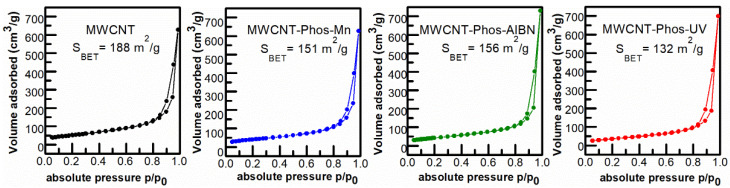
N_2_ adsorption–desorption isotherms and corresponding surface area of pristine and functionalized MWCNTs.

The surface area of the MWCNTs and functionalized MWCNTs were evaluated by N_2_ isothermal adsorption (Figure 8).

The adsorption–desorption isotherms represent a type IV isotherm, typical for multi-walled carbon nanotubes [78,79]. The observed hysteresis loop (0.8–0.99 p/p_0_) is associated with the mesoporous structure and corresponds well to the diameter of the used MWCNTs. After modification, the Brunauer–Emmett–Teller (BET) surface area of the MWCNT samples decreased from 188 to 132 m^2^/g for MWCNT–Phos–UV. A smaller decrease was observed for the two other samples, namely MWCNT–Phos–Mn and MWCNT–Phos–AIBN, of which thesurface areas were equal to 151 and 156 m^2^/g, respectively. The results obtained by all methods described above confirmed the highest functionalization level of MWCNT–Phos–UV sample, and the presence of contaminant in theform of MnO_2_ in the MWCNT–Phos–Mn sample. Therefore, the surface area of MWCNT–Phos–Mn cannot be compared with pristine MWCNTs or other samples as it resulted from an addition of two different materials. For the MWCNT–Phos–UV sample, Raman spectroscopy and TG analysis results demonstrated the most effective removal of amorphous carbon and probably the shortest and most defective MWCNTs, which caused a decrease in surface area.

## 4. Conclusions

Multiwalled carbon nanotubes were successfully functionalized in a free-radical phosphonylation reaction. Thermolysis of the P–H bond in diethyl phosphite was enhanced by two promoters: azo compound and single electron oxidant Mn(OAc)_3_. Photolysis of diethyl phosphite without any promoters also succeeded in homolytic cleavage of P–H bonds. It should be noted, that usage of Mn(OAc)_3_ was not suitable for the reactions of carbon nanotube functionalization, as the product of its decomposition contaminated the functionalized carbon material. Two other methods resulted in phosphonated carbon nanotubes, with a higher functionalization degree for the MWCNT–Phos–UV sample. UV illumination was found to be the most effective method for free-radical functionalization of the carbon nanotubes. Moreover, as compared to the other two protocols, the reaction time was substantially shorter and the temperature of the reaction medium did not exceed 40 °C. Therefore, this method can be used for temperature-sensitive materials. Functionalization with phosphonates significantly improve the thermal properties of MWCNTs and makes them promising materials as flame retardants additives.

The novel approach described here complements existing protocols for carbon nanomaterial functionalization. Moreover, it opens new possibilities for surface functionalization and the application of carbon nanotubes and other carbon nanostructures. 

## Figures and Tables

**Table 1:** Detailed conditions of the functionalization reactions.

Sample ID	MWCNTs	Radical Initiator	Phosphate Groups Source	Reaction Medium	Atm	Temp. (°C)	Time (h)
MWCNT- Phos-Mn	100 mg	90 mg Mn(OAc)_3_ ·2H_2_O	3 mL of (C_2_H_5_O)_2_P(O)H	3 mL of 1,2-dichlorobenzene	Ar	135	5
MWCNT- Phos-AIBN	100 mg	(150 mg + 150 mg) AIBN	3 mL of (C_2_H_5_O)_2_P(O)H	(C_2_H_5_O)_2_P(O)H	Ar	65	5
MWCNT- Phos-UV	100 mg	UV radiation (365–370 nm)	3 mL of (C_2_H_5_O)_2_P(O)H	(C_2_H_5_O)_2_P(O)H	Air	25	1

**Table 2:** Raman mode positions, linewidths (FWHM), and I_D_/I_G_ ratios for MWCNT, MWCNT–Phos–Mn, MWCNT–Phos–AIBN, and MWCNT–Phos–UV.

	D Band		G Band		
	Position (cm^−1^)	FWHM (cm^−1^)	Position (cm^−1^)	FWHM (cm^−1^)	I_D_/I_G_
MWCNT	1337	57	1596	69	1.88
MWCNT–Phos–Mn	1340	62	1597	77	1.53
MWCNT–Phos–AIBN	1334	65	1593	81	1.64
MWCNT–Phos–UV	1333	63	1593	72	1.49

**Table 3:** Comparison of the mass loss and differential thermal analysis of studied samples.

Sample Name	Temperature Range (°C)		
	40–450	450–600	600–850
	Mass Loss in % (DTG Peak Position (°C))		
MWCNT	0.87 (100, 270)	0.43 (-)	0.75 (-)
MWCNT–Phos–Mn	3.33 (166, 317)	0.97 (520)	1.63 (780)
MWCNT–Phos–AIBN	2.51 (148, 272)	0.34 (-)	1.20 (710)
MWCNT–Phos–UV	2.93 (158, 220)	0.29 (-)	1.43 (740)

## Data Availability

Data is contained within the article.
